# PKA and Ube3a regulate SK2 channel trafficking to promote synaptic plasticity in hippocampus: Implications for Angelman Syndrome

**DOI:** 10.1038/s41598-020-66790-4

**Published:** 2020-06-17

**Authors:** Jiandong Sun, Yan Liu, Guoqi Zhu, Caleb Cato, Xiaoning Hao, Li Qian, Weiju Lin, Rachana Adhikari, Yun Luo, Michel Baudry, Xiaoning Bi

**Affiliations:** 1 College of Osteopathic Medicine of the Pacific, Western University of Health Sciences, Pomona, CA 91766 USA; 2 Graduate College of Biomedical Sciences, Western University of Health Sciences, Pomona, CA 91766 USA; 3 College of Pharmacy, Western University of Health Sciences, Pomona, CA 91766 USA; 4 Key Laboratory of Xin’an Medicine, Ministry of Education, Anhui University of Chinese Medicine, Hefei, 230038 China

**Keywords:** Cellular neuroscience, Molecular neuroscience, Neurophysiology

## Abstract

The ubiquitin ligase, Ube3a, plays important roles in brain development and functions, since its deficiency results in Angelman Syndrome (AS) while its over-expression increases the risk for autism. We previously showed that the lack of Ube3a-mediated ubiquitination of the Ca^2+^-activated small conductance potassium channel, SK2, contributes to impairment of synaptic plasticity and learning in AS mice. Synaptic SK2 levels are also regulated by protein kinase A (PKA), which phosphorylates SK2 in its C-terminal domain, facilitating its endocytosis. Here, we report that PKA activation restores theta burst stimulation (TBS)-induced long-term potentiation (LTP) in hippocampal slices from AS mice by enhancing SK2 internalization. While TBS-induced SK2 endocytosis is facilitated by PKA activation, SK2 recycling to synaptic membranes after TBS is inhibited by Ube3a. Molecular and cellular studies confirmed that phosphorylation of SK2 in the C-terminal domain increases its ubiquitination and endocytosis. Finally, PKA activation increases SK2 phosphorylation and ubiquitination in Ube3a-overexpressing mice. Our results indicate that, although both Ube3a-mediated ubiquitination and PKA-induced phosphorylation reduce synaptic SK2 levels, phosphorylation is mainly involved in TBS-induced endocytosis, while ubiquitination predominantly inhibits SK2 recycling. Understanding the complex interactions between PKA and Ube3a in the regulation of SK2 synaptic levels might provide new platforms for developing treatments for AS and various forms of autism.

## Introduction

Ca^2+^-activated small conductance potassium (SK) channels play critical roles in the regulation of information flow along CNS circuits, and abnormal function of these channels has been implicated in a number of neurological and neuropsychiatric disorders, including epilepsy, ischemic neurodegeneration, schizophrenia, and autism^1–4^. The three subtypes of SK channels (SK1-3, aka, Kca2.1–2.3/KCNN1-3) exhibit differential but also overlapping regional and subcellular distributions in the CNS^5,6^. In hippocampal CA1 pyramidal neurons, SK2 channels are present in dendritic spines in close proximity to N-methyl-D-aspartate (NMDA) receptors^7,8^. It has recently been reported that the synaptic scaffold protein, MPP2 (membrane palmitoylated protein 2), is crucial for SK2 synaptic localization^9^. At CA1 excitatory synapses, SK2 channels are directly activated by Ca^2+^ entering through the NMDA receptors and repolarize the membrane, thereby inhibiting NMDA receptor function^10^. This local negative feedback loop between NMDARs and SK2 channels has been proposed to play a critical role in regulating neuronal excitability and in controlling the threshold for long-term potentiation (LTP) induction^8,11^. Blocking SK2 channels with the peptide toxin apamin facilitates hippocampal-dependent learning and memory in normal mice as well as in mouse models of Alzheimer’s disease^10,12–15^. Genetic deletion of SK2 channels, but not of SK1 or SK3 channels, abolishes the effect of apamin^16^, confirming the inhibitory effect of SK2 channels in learning and memory. On the other hand, LTP induction also regulates synaptic SK2 channel localization, as it triggers endocytosis of synaptic SK2 channels, which, in part, contributes to the enhancement of EPSPs at potentiated synapses^8^. This process is possibly due to protein kinase A (PKA)-mediated phosphorylation of three serine residues (Ser568–570) in the C-terminal domain of SK2 channels^8^. We previously found that synaptic levels of SK2 channels are regulated by another post-translational modification, namely Ube3a-mediated ubiquitination, and we showed that Ube3a-deficiency results in increased synaptic SK2 levels^17^. Of notice, recent research has indicated that activity-dependent down-regulation of SK channels may play important roles in learning and memory by regulating either intrinsic neuronal excitability or synaptic plasticity (see^18^ for a recent review).

Optimal CNS expression of UBE3A is crucial since its deficiency results in Angelman Syndrome (AS), while its over-expression increases the risk for autism^19,20^. Yet, the precise function of UBE3A in the CNS remains largely unknown. In AS mouse models, Ube3a deficiency leads to deficits in motor function, learning and memory, and social interactions^21–28^. Ube3a deficiency also results in impaired LTP induced by theta burst stimulation (TBS), but enhanced long-term depression (LTD) elicited by either low frequency stimulation (LFS)^17^ or application of agonists of group I mGluRs in field CA1 of hippocampus^29^. Ube3a deficiency causes enhanced postsynaptic SK2 levels, which shunts AMPAR-mediated depolarization, and effectively inhibits NMDAR activation, resulting in LTP impairment. While both PKA and Ube3a regulate synaptic SK2 levels, whether there are interactions between PKA- and Ube3a-mediated regulation of SK2 synaptic expression remains unknown. Likewise, whether PKA activation is beneficial in AS mice has not yet been tested. The current study provides evidence that synaptic SK2 levels are regulated by both PKA- and Ube3a-triggered endocytosis, and that PKA activation promotes LTP in hippocampal slices from AS mice. In addition, Ube3a-mediated SK2 ubiquitination inhibits its recycling to synaptic membranes after endocytosis. Finally, PKA-mediated SK2 phosphorylation increases Ube3a-mediated ubiquitination, and these two enzymes collaboratively maintain optimal synaptic SK2 levels.

## Results

### PKA activation restores TBS-induced LTP in hippocampal CA1 region in AS mice

**Figure 1:**
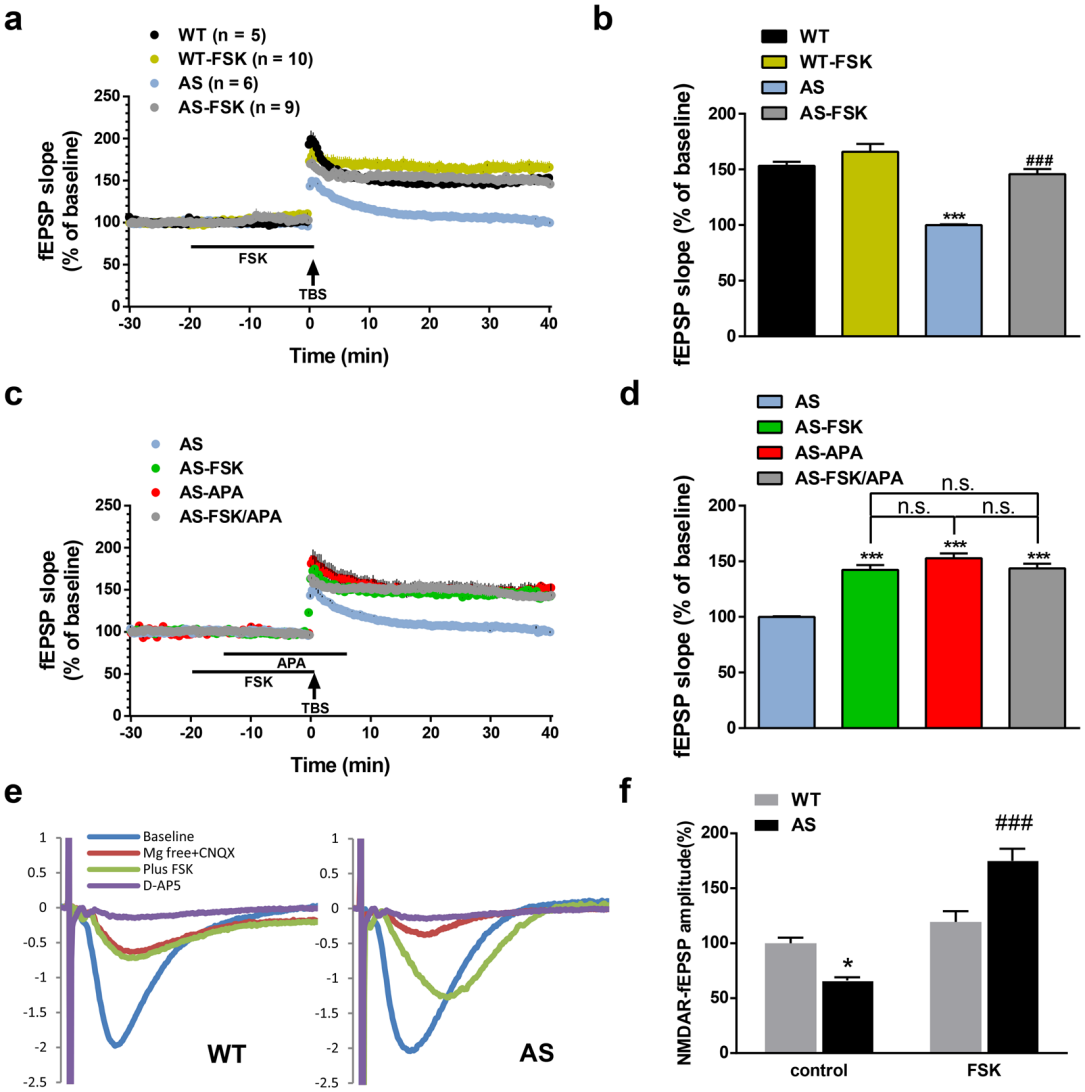
Effects of PKA activation on TBS-induced LTP and apamin-induced LTP rescue in hippocampus of AS mice. (**a,b**) Effect of forskolin (FSK) treatment on TBS-induced LTP in WT and AS mice. (**a**) Slopes of fEPSPs were normalized to the average values recorded during the first 10 min baseline. (**b**) Means ± S.E.M. of fEPSPs measured 40 min after TBS in different groups. N = 5–10 slices from 5–9 mice, p = 0.45, WT vs WT-FSK, p < 0.001, WT vs AS, p < 0.001, AS vs AS-FSK, two-way ANOVA with Tukey’s post-test. (**c,d**) Effects of FSK treatment on apamin (APA)-induced LTP rescue effect in AS mice. (**c**) Slopes of fEPSPs were normalized to the average values recorded during the first 10 min baseline. (**d**) Means ± S.E.M. of fEPSPs measured 40 min after TBS in different groups. N = 3-6 slices from 3-5 mice, p < 0.001, AS vs AS-FSK, p < 0.001, AS vs AS-APA, p < 0.001, AS vs AS-FSK/APA, p = 0.1788, AS-FSK vs AS-APA, p = 0.9929, AS-FSK vs AS-FSK/APA, p = 0.2739, AS-APA vs AS-FSK/APA, one-way ANOVA with Tukey’s post-test. n.s., not significant. (**e,f**) Effects of FSK treatment on NMDAR-mediated synaptic responses (_N_fEPSPs). (**e**) Representative traces. Scale bar, 0.5 mV/20 ms. (**f**) Quantification of _N_fEPSPs. Data are means ± SEM expressed as % of values in WT hippocampal slices before FSK treatment (WT control); p = 0.0488, WT control vs AS control, p < 0.001, AS control vs AS FSK (two-way ANOVA followed by Tukey test). Traces of AMPAR fEPSPs (blue lines) indicate that synaptic responses before initiation of _N_fEPSP recording are similar between AS and WT mice.

As previously reported by us and others^21,22,27,30,31^, TBS stimulation in hippocampal slices elicited LTP in field CA1 of WT mice but failed to do so in AS mice. We used a PKA activator, forskolin (FSK), to determine whether PKA activation could rescue LTP impairment in AS mice. Pre-incubation of hippocampal slices from AS mice with FSK (10 µM) for 20 min significantly improved TBS-induced LTP in AS mice, to levels indistinguishable from that in WT mice (Fig. 1a,b). Under these conditions, FSK application did not significantly affect LTP in WT mice; it also did not significantly modify baseline responses in mice of either genotype (Fig. 1a,b), which is consistent with our previous study^32^. Furthermore, when applied alone, apamin (20 nM), a selective SK2 channel blocker, or FSK, induced a similar degree of LTP enhancement in AS mice, and their combined application did not produce additive effects (Fig. 1c,d). These results suggest that PKA activation and apamin impact the same target, namely SK2 channels. We analyzed the effects of FSK treatment on NMDAR-mediated synaptic responses (_N_fEPSPs). _N_fEPSPs were isolated by bath application of Mg^2+^-free aCSF containing 6-cyano-7-nitroquinoxaline-2,3-dione (CNQX; 10 µM) to block AMPA-receptor-mediated synaptic responses. The NMDAR antagonist AP5 was used to verify that _N_fEPSPs were mediated by NMDARs under these conditions. _N_fEPSP amplitudes were significantly lower in AS mice, as compared to WT mice, and this decrease was reversed by FSK application (Fig. 1e,f). At this concentration, FSK did not significantly increase _N_fEPSP in WT mice (Fig. 1e,f). The effects of FSK were similar to those of apamin on _N_fEPSPs in AS and WT mice, as we previously reported^17^, which further supports the idea that PKA activation enhances LTP through removing SK2 channel-mediated inhibition of NMDAR-induced synaptic responses.

**Figure 2:**
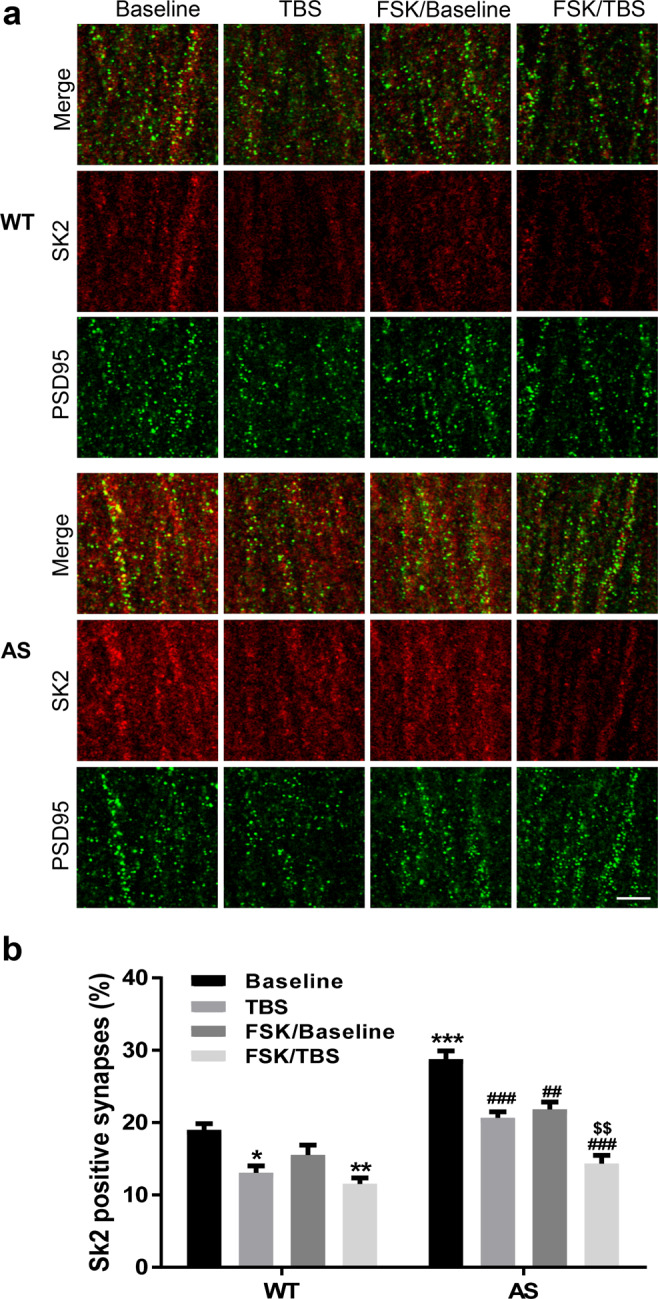
Effects of TBS and FSK treatment on the number of SK2-positive synapses in both WT and AS mice. (**a**) Representative images of SK2 (red) and PSD95 (green) co-immunostaining in the CA1 region of WT and AS mice after baseline recording (used as control) and 40 min after TBS applications with or without FSK treatment. Scale bar, 5 µm. (**b**) Quantitative results (mean ± SEM) of synapses dually labeled by anti-SK2 and anti-PSD95 antibodies in different experimental groups (p = 0.0163, WT Baseline vs WT TBS, p = 0.3209, WT Baseline vs WT FSK, p = 0.0022, WT Baseline vs WT FSK/TBS, p < 0.001, WT baseline vs AS Baseline, p = 0.001, AS Baseline vs AS TBS, p = 0.0045, AS Baseline vs AS FSK, p < 0.001, AS Baseline vs AS FSK/TBS, p = 0.0098, AS TBS vs AS FSK/TBS, n = 3 slices from 3 mice/group, two-way ANOVA followed by Tukey test).

We used immunohistochemistry to analyze the subcellular distribution of SK2 channels under various experimental conditions. Quantitative image analysis showed that TBS stimulation induced a significant reduction in the density of SK2-immunopositive PSD95-labeled synapses in both WT and AS mice (Fig. 2). In hippocampal slices from AS mice, FSK application resulted in a significant reduction in the density of SK2-immunopositive synapses under basal conditions and a further reduction following TBS application (Fig. 2), which could account for the facilitating effect of PKA activation on TBS-induced LTP in AS mice (Fig. 1). In WT mice, a significant reduction in the density of SK2-immunopositive synapses was only observed following TBS application; FSK treatment alone did not significantly reduce the density of SK2-immunopositive synapses, nor did it change TBS-induced reduction. These results are consistent with and provide possible molecular basis for the electrophysiological results shown in Fig. 1. They also suggest that PKA-induced SK2 endocytosis occurs mainly following tetanic stimulation, while basal synaptic SK2 levels are predominantly regulated by Ube3a-mediated ubiquitination.

### Role of PKA-mediated SK2 channel internalization in repeated TBS-induced LTP in AS mice

**Figure 3:**
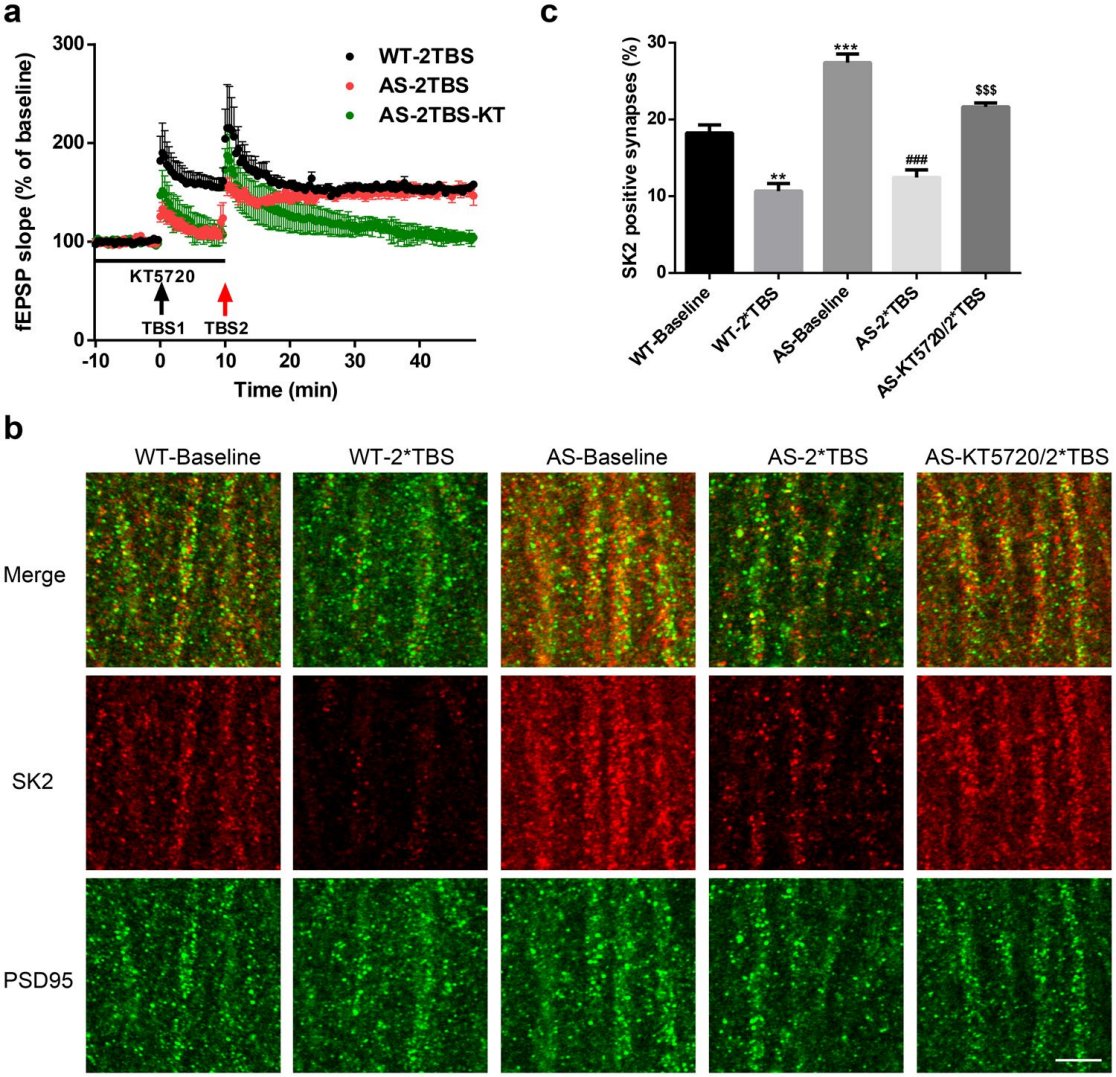
Effects of PKA inhibition on 2xTBS-induced LTP and synaptic SK2 levels in hippocampal slices of AS mice. (**a**) PKA inhibition with KT5720 blocked 2xTBS-induced LTP in AS mice. (**b**) Representative images of SK2 (red) and PSD95 (green) co-immunostaining in the CA1 region of WT and AS mice after baseline recording (used as control) and 2xTBS applications with or without KT5720 treatment. Scale bar, 5 µm. (**c**) Quantitative results (mean ± SEM) of synapses dually labeled by anti-SK2 and anti-PSD95 antibodies in different experimental groups (p = 0.0017, WT Baseline vs WT 2xTBS, p < 0.001, WT Baseline vs AS Baseline, p < 0.001, AS Baseline vs AS 2xTBS, p < 0.001, AS 2xTBS vs AS KT5720/2xTBS, n = 3 slices from 3 mice/group, one-way ANOVA followed by Tukey test).

We previously reported that, although one TBS did not induce LTP in hippocampal slices from AS mice, a second TBS (TBS2) applied 10 min after the first TBS (TBS1) could induce LTP^17^, which was confirmed in the current study (Fig. 3a). TBS2-induced LTP was occluded by apamin pre-treatment, suggesting that TBS1-triggered internalization of SK2 proteins facilitates LTP induction by TBS2^17^. Previous research has shown that LTP induction induces PKA-dependent SK2 channel internalization^8^. We therefore tested the effect of a PKA inhibitor, KT5720, on LTP induction by TBS2 in AS mice. Application of KT5720 10 min before TBS1 and maintained for another 10 min blocked TBS2-induced LTP in AS mice (Fig. 3a), which could be due to the inhibition of PKA-dependent SK2 channel internalization by TBS1. Indeed, double immunostaining of SK2 and PSD95 revealed that the density of SK2-immunopositive synapses was significantly reduced after TBS in both WT and AS mice, while pre-incubation with KT5720 blocked TBS-induced SK2 reduction in AS mice (Fig. 3b,c).

### Ube3a-mediated SK2 ubiquitination inhibits SK2 targeting to recycling endosomes

**Figure 4:**
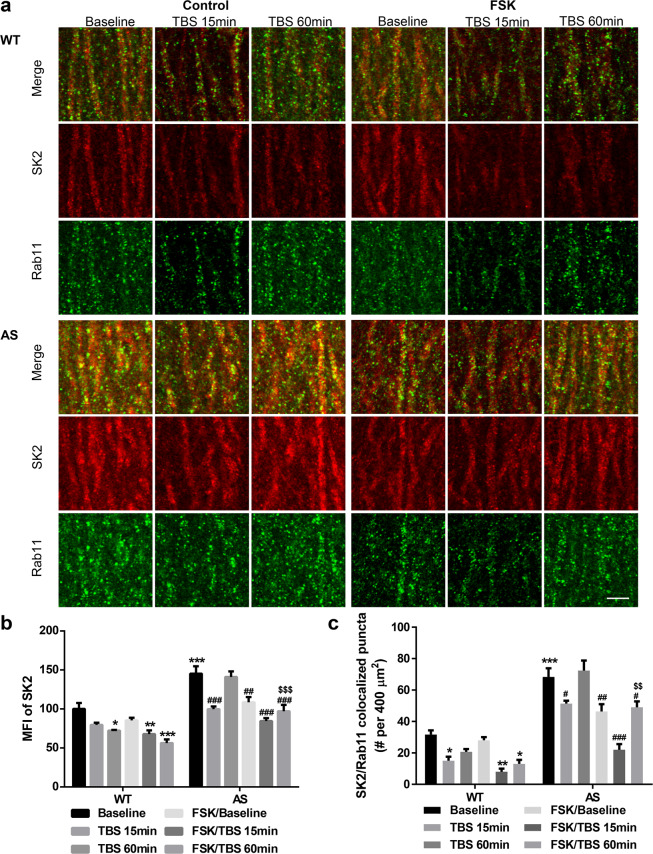
SK2 and Rab11 co-immunostaining in hippocampal CA1 region of WT and AS mice at different time points after baseline recording (used as control) and TBS applications with or without FSK treatment. (**a**) Representative images of SK2 (red) and Rab11 (green) co-immunostaining. Scale bar, 5 µm. (**b**) Quantitative results (mean ± SEM) of mean fluorescent intensity (MFI) of SK2 in different experimental groups (p = 0.0267, WT Baseline vs WT TBS 60 min, p = 0.0077, WT Baseline vs WT FSK/TBS 15 min, p < 0.001, WT Baseline vs WT FSK/TBS 60 min, p < 0.001, WT baseline vs AS Baseline, p < 0.001, AS Baseline vs AS TBS 15 min, p = 0.0020, AS Baseline vs AS FSK/Baseline, p < 0.001, AS Baseline vs AS FSK/TBS 15 min, p < 0.001, AS Baseline vs AS FSK/TBS 60 min, p < 0.001, AS TBS 60 min vs AS FSK/TBS 60 min, n = 3 slices from 3 mice/group, two-way ANOVA followed by Tukey test). (**c**) Quantitative results (mean ± SEM) of puncta dually labeled by anti-SK2 and anti-Rab11 antibodies in different experimental groups (p = 0.0361, WT Baseline vs WT TBS 15 min, p = 0.0014, WT Baseline vs WT FSK/TBS 15 min, p = 0.0149, WT Baseline vs WT FSK/TBS 60 min, p < 0.001, WT baseline vs AS Baseline, p = 0.0313, AS Baseline vs AS TBS 15 min, p = 0.0032, AS Baseline vs AS FSK/Baseline, p < 0.001, AS Baseline vs AS FSK/TBS 15 min, p = 0.011, AS Baseline vs AS FSK/TBS 60 min, p = 0.0017, AS TBS 60 min vs AS FSK/TBS 60 min, n = 3 slices from three mice/group, two-way ANOVA followed by Tukey test). See also Supplementary Fig. 1.

We previously showed that the ability of TBS2 to restore LTP in AS mice decreased at 60 min after TBS1, although TBS2 in WT mice further enhanced LTP magnitude at this time point^17^. It has been previously shown that synapses that develop LTP have decreased SK2 levels and that SK2 levels return to control levels by 2 h after LTP induction^33^. In agreement with the literature, levels of synaptic SK2 in hippocampal slices from WT mice were slightly reduced 15 min after TBS, which was followed by a larger and significant reduction 60 min after TBS (Fig. 4 and Supplementary Fig. 1). In contrast, in hippocampal slices from AS mice, synaptic SK2 levels were significantly reduced at 15 min after TBS but returned to baseline level 60 min after TBS (Fig. 4a,b and Supplementary Fig. 1). We next performed co-immunostaining of SK2 with Rab11, a recycling endosome marker, to analyze the dynamics of SK2 recycling back to synaptic membranes. The density of SK2/Rab11 dually labeled puncta in WT mice significantly decreased 15 min after TBS, and remained lower than baseline 60 min after TBS, although the difference at this time was not statistically significant (Fig. 4a,c, and Supplementary Fig. 1). In AS mice, the density of SK2/Rab11 dually labeled puncta also decreased 15 min after TBS, but returned to baseline levels 60 min after TBS. These results are consistent with the notion that SK2 channels are recycled to synaptic membranes much faster in AS than in WT mice. FSK application before TBS further reduced synaptic SK2 levels and the density of SK2/Rab11 dually labeled puncta in both WT and AS mice 60 min after TBS. FSK application reduced basal (without TBS) synaptic SK2 levels and the density of SK2/Rab11 dually labeled puncta only in AS mice (Fig. 4 and Supplementary Fig. 1). Interestingly, increased SK2 staining in AS mice under basal condition was associated with increased co-localization with Rab11, which suggests that the lack of Ube3a favors SK2 channel recycling to synaptic membranes. In addition, triple staining of SK2, PSD95, and Rab11 confirmed these results, and revealed obvious SK2/PSD95/Rab11-positive puncta in AS mice under basal condition as well as 60 min after TBS (Supplementary Fig. 1).

### Mutation of the PKA phosphorylation sites in SK2 C-terminal domain alters SK2 ubiquitination and endocytosis

**Figure 5:**
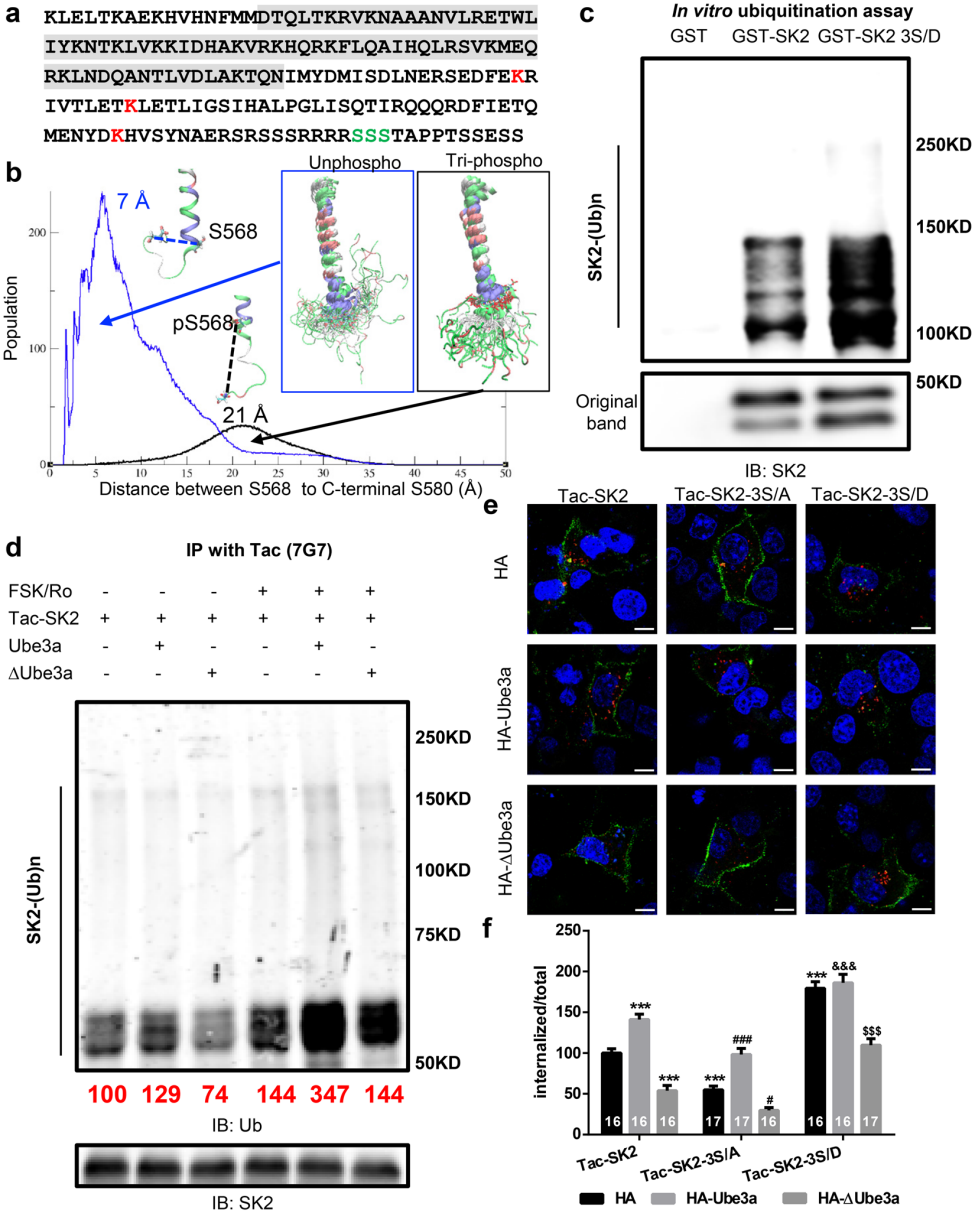
Effects of phosphorylation of SK2 on Ube3a-mediated SK2 ubiquitination and SK2 endocytosis. (**a**) Potential phosphorylation (green SSS) and ubiquitination sites (red Ks) in the C-terminal domain of SK2 channel (shaded sequence indicates calmodulin binding domain). (**b**) Distribution of distance between phosphorylation site S568 and C-terminal residue S580 obtained from 100 ns molecular dynamics simulation of SK2 C-terminal helix in explicit solvent. Blue is the distribution curve without phosphorylation, black is that with phosphorylation on serine 568-570. The representative conformation of the helix with the peak distance of 7 Å for unphosphorylated system and 21 Å for tri-phosphorylated system are shown next to each peak. The overlay of the helix backbone conformations from every 2 ns are shown for each system. Serine 568-570 are shown in licorice. The backbones are colored by residue types: positively charged in blue, negatively charged in red, polar in green, and nonpolar in white. (**c**) *In vitro* ubiquitination of SK2 and its phosphomimetic mutant SK2-3S/D by recombinant Ube3a. Reaction products were analyzed by Western blot with SK2 antibodies. (**d**) Representative images and quantitative analysis (shown as numbers in red; normalized to the Tac-SK2 group set to 100%; N = 3 independent experiments) of Western blots labeled with ubiquitin (Ub) and SK2 antibodies. Samples from COS-1 cells co-transfected with Tac-SK2 plus Ube3a or ∆Ube3a and treated with either DMSO or forskolin (FSK) and Ro 20-1724 were immunoprecipitated using mouse anti-Tac antibodies and probed with indicated antibodies. (**e,f**) Effects of Ube3a overexpression and S-A or S-D mutations on SK2 surface expression and endocytosis. (**e**) Representative images of internalized (red) or surface-expressed (green) Tac-SK2, 3S/A, and 3S/D in COS-1 cells co-transfected with HA (control; top), HA-Ube3a (middle), or HA-∆Ube3a (bottom). Scale bar, 10 µm. (**f**) Quantitative analysis of images in e. Data are expressed as mean ± SEM. p < 0.001 for Tac-SK2/HA vs Tac-SK2/HA-Ube3a, Tac-SK2/HA vs Tac-SK2/HA-∆Ube3a, Tac-SK2-3S/A/HA vs Tac-SK2-3S/A/HA-Ube3a, Tac-SK2-3S/D/HA vs Tac-SK2-3S/D/HA-∆Ube3a, Tac-SK2/HA vs Tac-SK2-3S/A/HA, Tac-SK2/HA vs Tac-SK2-3S/D/HA, Tac-SK2/HA-Ube3a vs Tac-SK2-3S/D/HA-Ube3a; p = 0.0302 Tac-SK2-3S/A/HA vs Tac-SK2-3S/A/HA-∆Ube3a; two-way ANOVA with Tukey post hoc analysis. N = cells is indicated in each column and from at least 3 independent experiments. See also Supplementary Fig. 2.

To investigate whether PKA-mediated phosphorylation of residues 568–570 (green SSS in Fig. 5a) alters the conformation of SK2 C-terminal domain, we constructed in silico a SK2 C-terminal region (residue 526–580) using homology modeling tool and performed 100 nanoseconds (ns) molecular dynamics simulations of both unphosphorylated and phosphorylated (pSer568–570) C-terminal domain in explicit solvent. We found that the helix structure was stable under both simulations. The main difference consisted in the overall conformation of the tail of the C-terminal domain (571–580). Figure 5b shows the distribution of the distances between the first phosphorylation site (S568) and the last residue on the C-terminal domain (S580) over 5000 snapshots evenly extracted from 100 ns trajectories. The tail of the C-terminal domain, albeit quite flexible, sampled very different conformational spaces under the two conditions; the peak distance shifted from 7 Å to 21 Å when Ser568–570 were phosphorylated. It is therefore possible that the change in flexibility of the C-terminal domain resulting from PKA-mediated phosphorylation alters Ube3a-mediated SK2 channel ubiquitination.

In order to directly test the effect of phosphorylation at residues Ser568–570 of SK2 on Ube3a-mediated ubiquitination, we generated GST-SK2 C-terminal conjugates with or without the three serine residues mutated to aspartate (GST-SK2 3S/D) (phosphomimetic)^34^. We then performed *in vitro* ubiquitination assay to determine ubiquitination levels of GST (used as a negative control), GST-SK2, and GST-SK2 3S/D using the E6AP/UBE3A assay kit. The ubiquitination level of GST-SK2 3S/D was markedly increased, as compared with that of GST-SK2 (Fig. 5c). The effect of PKA-mediated phosphorylation of SK2 on Ube3a-mediated ubiquitination was then tested using COS-1 cells transfected with a chimeric construct (Tac-SK2) containing the N-terminal and transmembrane domains of Tac, a constitutively expressed membrane protein^35^, and the SK2 C terminus. To activate PKA in heterologous cells, we used a combination of FSK and Ro 20–1724 (a phosphodiesterase inhibitor), a protocol previously used to show that PKA-mediated phosphorylation induces SK2 endocytosis^36^, to treat COS-1 cells expressing Tac-SK2 with HA-empty vector, WT-Ube3a, or ΔUbe3a (an inactive form of Ube3a with a mutation in its catalytic site, Ube3a-C833A)^37^. We then performed immunoprecipitation assay with Tac antibody; co-transfection with Ube3a increased, while co-transfection with ∆Ube3a decreased SK2-C ubiquitination, as compared to the endogenous Ube3a group (Fig. 5d). Of note, PKA activation further increased ubiquitination of SK2 in all three groups with the Ube3a-transfected group showing the highest levels of ubiquitinated SK2 (Fig. 5d).

To investigate the effects of SK2 phosphorylation on surface expression and internalization, we performed detailed analyses in COS-1 cells using Tac-SK2. Ser568–570 were mutated to alanine (3S/A-SK2; non-phosphorylatable) or aspartate (3S/D-SK2; phosphomimetic) in Tac-SK2, and COS-1 cells were co-transfected with Tac-SK2 or its mutants with HA-empty vector, WT-Ube3a, or ΔUbe3a. Endocytosis analysis experiments (see Methods) showed that the number of internalized SK2 puncta was markedly reduced in 3S/A-SK2 expressing cells but increased in 3S/D-SK2 expressing cells, as compared to those expressing Tac-SK2 (Fig. 5e,f). Co-expression with WT-Ube3a significantly increased, while co-expression with ∆Ube3a significantly reduced Tac-SK2 internalization (Fig. 5e,f). Interestingly, WT-Ube3a had similar effects on the non-phosphorylatable mutant 3S/A-SK2 as on Tac-SK2 (Fig. 5e,f). Increased internalization of 3S/D-SK2 was markedly reduced by the expression of ΔUbe3a, which exhibits dominant negative property (Fig. 5e,f). Expression of WT-Ube3a did not further enhance 3S/D-SK2 internalization (Fig. 5e,f). To understand the kinetics of SK2 internalization, we also performed co-immunolocalization with the early endosome marker EEA1, and the late endosome/lysosome marker LAMTOR4. Most of the internalized SK2 puncta were co-localized with EEA1, while only a few co-localized with LAMTOR4 (Supplementary Fig. 2).

### PKA activation increases Ube3a-mediated SK2 ubiquitination in Ube3a transgenic mice, resulting in reduced synaptic SK2 levels

**Figure 6:**
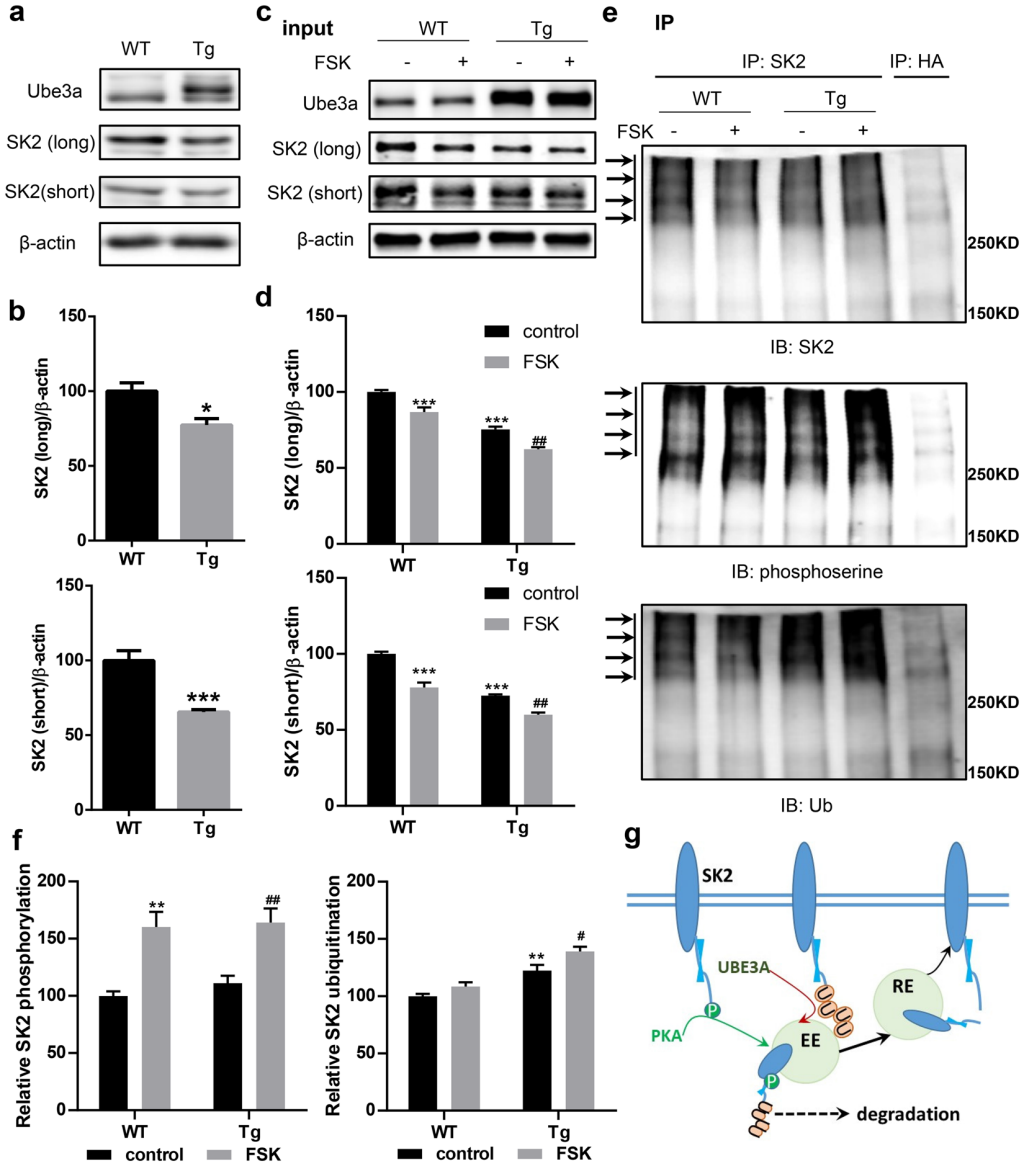
SK2 expression in hippocampus of Ube3a transgenic (Tg) and wild-type (WT) mice. (**a,b**) Western blot analysis of SK2 levels in crude synaptosomal fractions (P2) from hippocampus of WT and Tg mice. Optical densities of the SK2 band are normalized to those of β-actin. Data are expressed as % of values in WT mice and shown as means ± SEM of 5 mice from at least 3 litters; p = 0.0122 for SK2-long, p = 0.001 for SK2-short (unpaired t test). (**a**) representative Western blot images; (**b**) quantitative analysis of blots in a. (**c,d**) Effects of acute forskolin (FSK) treatment on SK2 levels in hippocampus slices of WT and Tg mice. (**c**) representative Western blot images; (**d**) quantitative analysis of blots in c. N = 6 independent experiments, for SK2-long, p < 0.001 WT/control vs WT/FSK, p < 0.001 WT/control vs Tg/control, p = 0.001 Tg/control vs Tg/FSK; for SK2-short, p < 0.001 WT/control vs WT/FSK, p < 0.001 WT/control vs Tg/control, p = 0.0011 Tg/control vs Tg/FSK, two-way ANOVA with Tukey’s post-test. (**e,f**) Effects of acute FSK treatment on ubiquitination and phosphorylation of SK2 in hippocampus slices of WT and Tg mice. (**e)** Representative Western blot images. Immunoprecipitation was performed with anti-SK2 antibodies, and Western blots were labeled with anti-SK2, -phosphoserine, and -ubiquitin (Ub) antibodies. Arrows indicate ubiquitinated and/or phosphorylated SK2 proteins. (**f**) Quantitative analysis of blots in e. N = 5 independent experiments. Left panel, p = 0.0025 WT/control vs WT/FSK, p = 0.0073 Tg/control vs Tg/FSK; Right panel, p = 0.004 WT/control vs Tg/control, p = 0.0335 Tg/control vs Tg/FSK, two-way ANOVA with Tukey’s post-test. (**g)** Model for SK2 channel regulation by Ube3a-ubiquitination and PKA-phosphorylation. PKA phosphorylation and Ube3a ubiquitination result in SK2 endocytosis in early endosomes (EE). Ubiquitinated SK2 does not recycle back to synaptic membranes through recycling endosomes (RE). PKA and Ube3a jointly regulate synaptic SK2 levels; phosphorylation facilitates ubiquitination.

We previously showed that the increase in synaptic SK2 levels in AS mice was due to the lack of Ube3a-mediated ubiquitination. In contrast, SK2 levels were significantly lower in crude synaptosomal (P2) fractions of Ube3a-overexpressing transgenic (Tg) mice than in those from WT mice (Fig. 6a,b), further supporting the idea that SK2 is a substrate of Ube3a. Crude membrane fractions enriched in synaptic proteins were prepared from acute hippocampal slices of Tg or WT mice following treatment with vehicle or FSK (50 µM). Western blots showed that PKA activation significantly decreased SK2 levels in both WT and Tg mice (Fig. 6c,d). Of note, two isoforms of SK2 differing only in the length of the N-terminal domain^7^, long-form and short-form SK2, showed similar changes under these conditions. Immunoprecipitation of P2 fractions with SK2 antibodies from either WT or Tg hippocampal slices with or without FSK treatment confirmed that SK2 was phosphorylated and that its phosphorylation was increased in WT and Tg mice following FSK treatment (Fig. 6e, middle panel; Fig. 6f, left panel). SK2 was also ubiquitinated, and its ubiquitination was significantly higher in Tg mice, as compared to WT mice (Fig. 6e, bottom panel; Fig. 6f, right panel). Notably, FSK treatment further increased SK2 ubiquitination in Tg mice, but it did not change that in WT mice (Fig. 6e, bottom panel; Fig. 6f, right panel), providing *in situ* evidence that SK2 phosphorylation enhances its ubiquitination. These results therefore are in good agreement with those obtained in COS cells.

## Discussion

Our results indicate that both PKA activation and SK2 channel inhibition can reverse TBS-induced LTP impairment in hippocampal slices from Ube3a-deficient mice and that the effects of these two manipulations are mutually occlusive, indicating that they both target SK2 channels. Both PKA and Ube3a regulate synaptic SK2 levels by stimulating SK2 endocytosis. PKA activation during LTP induction results in SK2 phosphorylation followed by rapid internalization. Ube3a-mediated SK2 ubiquitination not only targets it into early endosomes, but also inhibits its sorting to recycling endosomes, which favors SK2 channel degradation. Consequently, the lack of Ube3a in AS mice results in increased levels of SK2 channels in recycling endosomes and synapses. Both *in vitro* and *in vivo* experiments revealed that PKA-mediated SK2 phosphorylation increases Ube3a-mediated ubiquitination and suppression of SK2 recycling to synaptic membranes. Together, these results indicate that PKA and Ube3a can independently and collaboratively regulate synaptic SK2 levels, and that the interaction between these regulatory mechanisms depends on neuronal activity history.

### PKA activation reverses TBS-induced LTP impairment in hippocampal slices from AS mice by removing SK2-mediated brake on NMDAR

We previously showed that Ube3a ubiquitinates SK2 channels and reduces their synaptic levels, and that the lack of this regulation in AS mice contributes to TBS-induced LTP impairment and reduces learning performance^17^. The present study provides the first evidence that PKA activation can restore LTP in AS mice by removing SK2-mediated inhibition of NMDAR. Several results support this conclusion. First, FSK application in hippocampal slices enhanced NMDAR-mediated synaptic responses and restored LTP in AS mice while it had no effect in WT mice; these effects were similar to those of apamin we reported in our previous study^17^. The effect of FSK on LTP was occluded by apamin, further supporting the idea that FSK targets SK2 channels. Second, we showed that a second TBS applied 10 min after a first TBS could induce LTP in AS mice, and this effect was blocked by application of a PKA inhibitor, KT5720. We postulate that, although one TBS can induce SK2 endocytosis in AS mice in a PKA-dependent manner, the reduction of synaptic SK2 levels it elicits is not sufficient to remove NMDAR inhibition. These results suggest that the reduction in SK2 levels must reach a certain “threshold” in order to release NMDAR inhibition and for TBS-LTP to occur. This interpretation is supported by results indicating that FSK enhances NMDAR-mediated synaptic responses and LTP only in AS mice, but not in WT mice. As mentioned earlier, electrophysiological results correlated well with the quantitative immunocytochemical results, which indicated that under basal condition, FSK application reduced synaptic SK2 levels in AS mice to levels similar to those found in WT mice. These results could account for the apparent discrepancy with previous findings that both FSK and apamin facilitate LTP in WT mice^8,38^ and rats^39^; under our conditions, FSK did not significantly alter synaptic SK2 levels and apamin did not modify NMDAR-mediated synaptic responses in WT mice. Perhaps the lack of Ube3a-mediated ubiquitination in AS mice alters SK2 regulation and makes them more susceptible to PKA-dependent regulation.

In contrast to the lack of effects on basal levels of synaptic SK2 and NMDAR-mediated synaptic responses in WT mice, FSK application did enhance TBS-induced decrease in synaptic SK2 levels in WT mice as well, which correlates with FSK-induced slight increase in LTP in WT mice. These results indicate that PKA activation is necessary but not sufficient for triggering significant SK2 endocytosis, and that some other factors triggered by TBS contribute to SK2 endocytosis. PKA activation during LTP induction has been shown to phosphorylate AMPARs and to facilitate their membrane insertion, which in turn facilitates SK2 internalization^33^. Activation of CamKII and ERK has also been shown to play critical roles in LTP induction and consolidation, including promoting AMPAR synaptic insertion^40,41^. Along this line, we have shown that restoring LTP in AS mice is associated with increased AMPAR synaptic expression^27^; whether SK2 endocytosis is also mechanistically linked to AMPAR insertion in AS mice remains to be determined.

### Ube3a-dependent ubiquitination hinders SK2 recycling to synaptic membranes

Synaptic membrane proteins undergo dynamic insertion and internalization; endocytosed membrane receptors/channels are either recycled back to membranes or degraded through the ubiquitin-proteasome and/or the late-endosome/lysosome systems. It has been previously shown that synapses that develop LTP exhibit decreased SK2 levels and that this decrease lasts for more than 2 h after LTP induction^33^. Rab11 has been shown to play critical roles in recycling membrane proteins, including AMPARs undergoing constitutive recycling and during synaptic plasticity-induced recycling [^42^; also see^43^, for a recent review]. We showed here for the first time that Rab11 is also associated with SK2 trafficking after TBS. In the absence of Ube3a-mediated ubiquitination, membrane SK2 channels undergo faster shuttling between PSD-95-positive synaptic contacts and recycling endosomes, as well as a faster (by 60 min) recovery to baseline levels after TBS stimulation (Fig. 4). Thus, Ube3a deficiency increases synaptic SK2 levels not only by reducing SK2 endocytosis but also by enhancing its recycling to synaptic membranes. How Ube3a deficiency results in faster Rab11-mediated recycling of SK2 is not clear. This effect might involve adaptor and motor proteins, such as myosin Vb and the endosomal adaptor Rab11-FIP2^43^. It is also conceivable that Ube3a-mediated ubiquitination of SK2 channels targets them to degradation pathways instead of recycling pathways. It is interesting that FSK treatment further reduced both SK2 levels and the density of SK2/Rab11 dually labeled puncta in AS mice 60 min after TBS (Fig. 4), suggesting that PKA activation not only facilitates TBS-induced SK2 internalization, but also inhibits SK2 recycling to synaptic membranes. It is tempting to speculate that PKA-mediated inhibition of SK2 recycling may be linked to its facilitation of SK2 ubiquitination. Of note, FSK treatment under our conditions did not affect basal levels (without TBS) of SK2/Rab11 dually labeled puncta in WT mice but it did reduce them in AS mice. These results suggest that the lack of Ube3a-mediated ubiquitination alters the regulation of SK2 trafficking dynamics.

### Ube3a-dependent SK2 ubiquitination and PKA-dependent SK2 phosphorylation can occur independently and collaboratively

Previous results demonstrated that the subcellular localization of SK2 was regulated by phosphorylation through the PKA pathway or by ubiquitination through the Ube3a pathway^17,36^. Our results showed that SK2 with the S to D phosphomimetic mutation, exhibited reduced surface expression and increased endocytosis, while the S to A mutation resulted in the opposite effects. Furthermore, GST-SK2 3S/D conjugates exhibited higher levels of ubiquitination in the Ube3a assay (Fig. 5c), suggesting that SK2 phosphorylation in the C-terminal domain increases its ubiquitination in the same region; we previously showed that Ube3a ubiquitinates SK2 at residues K506,K514,K550^17^. The effect of PKA-mediated phosphorylation of SK2 on Ube3a-mediated ubiquitination was also confirmed in heterologous cells. Co-transfection experiments of WT Ube3a or dominant negative Ube3a mutants together with phosphorylation site mutants showed that SK2 phosphorylation is not necessary for, but facilitates, Ube3a-induced SK2 internalization (Fig. 5e,f). It is noteworthy that although co-transfection with mutant Ube3a significantly reduced SK2 3S/D internalization, co-transfection with WT Ube3a did not further enhance its internalization; this finding is probably due to the fact that, in the presence of endogenous Ube3a, 3S/D-SK2 may already undergo maximal endocytosis.

Crosstalk between phosphorylation and ubiquitination exists ubiquitously from plants to animals and plays critical roles at fine tuning signal transduction. These two posttranslational modifications can either occur independently or one can function as a prerequisite for the other, and influence each other positively, negatively or neutrally. For example, tyrosine phosphorylation of EGFR upon its binding with EGF has been shown to facilitate its ubiquitination by the Ring domain-containing E3 ligase Cbl and its subsequent degradation^44,45^. Furthermore, phosphorylation of E3 ligase can affect its binding and/or catalytic activity toward a substrate, while ubiquitination of a kinase can also affect it turnover/stability. Indeed, a recent study revealed that PKA phosphorylates Ube3a at residue T485 and inhibits its ligase activity^46^. We showed that PKA activation increased TBS-induced SK2 internalization in both WT and AS mice and that phosphorylation facilitates SK2 ubiquitination, although phosphorylation is not a prerequisite for ubiquitination. Together with results from the experiments with mutations of SK2 phosphorylation sites, our findings indicate that PKA-mediated phosphorylation of SK2, rather than of Ube3a, is critical for the regulation of SK2 synaptic levels. Finally, results with Ube3a-overexpressing transgenic mice showed that increased Ube3a expression results in increased SK2 ubiquitination and decreased membrane SK2 levels, which confirms the critical role of Ube3a in SK2 regulation. PKA activation increased SK2 phosphorylation similarly in WT and Tg mice, and further increased SK2 ubiquitination in Tg mice, which indicates that phosphorylation increases ubiquitination of SK2 in hippocampal slices as well, while ubiquitination may not affect phosphorylation.

In conclusion, both PKA-mediated phosphorylation and Ube3a-mediated ubiquitination regulate synaptic levels of SK2 channels, which are emerging as a key player in coordinating Ca^2+^ signaling, dendritic/neuronal excitability, synaptic plasticity, and rhythmic activity in CNS^8,10,47,48^. Both PKA activation and Ube3a independently regulate SK2 trafficking and synaptic membrane SK2 levels, but PKA-mediated phosphorylation facilitates Ube3a-mediated ubiquitination. This mechanism provides an opportunity to correct SK2-mediated impairment in synaptic plasticity and learning and memory in AS through the activation of PKA. Since Ube3a has been implicated in Angelman Syndrome and autistic spectrum disorder, and since SK2 channels are widely distributed and play critical roles in both CNS and peripheral systems, a better understanding of SK2 regulation by Ube3a and PKA could provide broad therapeutic applications for a variety of diseases.

## Methods

### Animals

Animal experiments were conducted in accordance with the principles and procedures of the National Institutes of Health Guide for the Care and Use of Laboratory Animals. All protocols were approved by the Institutional Animal Care and Use Committee of Western University of Health Sciences. Original Ube3a mutant (AS) mice were obtained from The Jackson Laboratory, strain B6.129S7-*Ube3a*^*tm1Alb*^/J (stock No. 016590), and a breeding colony was established, as previously described^23^. The FLAG-Ube3a BAC transgenic (Tg) mice were generated by Dr. Matthew P. Anderson’s lab^49^, and purchased from The Jackson Laboratory, strain FVB/NJ-Tg(Ube3a)1Mpan/J (stock No. 019730). Wild-type (WT) and Tg (Ube3a 1xTg) mice were obtained in-house through breeding of hemizygous mice with WT (noncarrier) mice from the colony or with FVB/NJ inbred mice (Stock No. 001800). Mouse genomic DNA was isolated from tail biopsies and genotype was determined by PCR following the protocol provided by The Jackson Laboratory. In all experiments male AS or Tg mice aged between 2–4 months were used. Control mice were age-matched, male, wild-type littermates. Mice, housed in groups of two to three per cage, were maintained on a 12-h light/dark cycle with food and water ad libitum.

### Cell lines, DNA constructs, and transfection

COS-1 cells were grown in DMEM supplemented with 10% (vol/vol) fetal bovine serum (FBS) (Invitrogen). Expression constructs encoding HA-tagged wild-type Ube3a, HA-tagged catalytically inactive mutant Ube3a-C833A (substitution of Cys-833 by Ala) were gifts from Peter Howley (Addgene plasmid # 8648 and 8649)^50^. Expression construct encoding Tac-SK2 C-terminus was generated as previously described^17^. GST-SK2 carboxyl-terminal construct was a gift from Dr. John Adelman^51^. Constructs with S-A or S-D mutations were generated from Tac-SK2 or GST-SK2 by site-directed mutagenesis (Agilent).

For transient expression, cells were transfected with the respective constructs by lipofection (Lipofectamine 2000; Invitrogen) according to the manufacturer’s instructions.

### Reagents

The following primary antibodies were used: Tac7G7 (7G7B6, ATCC, HB-8784), SK2 (Alomone, APC-028), SK2 (Alomone, AGP-045), Ube3a (Sigma, E8655), Ube3a (Bethyl Laboratories, A300–351A), PSD95 (Invitrogen, MA1–045), Rab11 (abcam, ab95375), Ubiquitin (Ub, abcam, ab7780), Ub (Santa Cruz, sc-9133), Phosphoserine (Millipore, ab1603), HA (Sigma, H6908), EEA1 (abcam, ab2900), LAMTOR4 (Cell signaling technology, 12284), and β-actin (Sigma, A5441). All secondary antibodies for Western blots were obtained from LI-COR, and for immunofluorescence Alexa-488, −594, and −633 conjugated secondary antibodies were obtained from Invitrogen. Forskolin was obtained from Sigma; apamin was obtained from Millipore; Ro 20–1724 was purchased from Santa Cruz, and KT5720, CNQX, and D-AP5 were purchased from Tocris.

### Acute hippocampal slice preparation

Adult male mice (2–4-month-old) were anesthetized with gaseous isoflurane and decapitated. Brains were quickly removed and transferred to oxygenated, ice-cold cutting medium (in mM): 124 NaCl, 26 NaHCO_3_, 10 glucose, 3 KCl, 1.25 KH_2_PO_4_, 5 MgSO_4_, and 3.4 CaCl_2_. Hippocampal transversal slices (400 μm-thick) were prepared using a McIlwain-type tissue chopper and transferred to i) an interface recording chamber and exposed to a warm, humidified atmosphere of 95%O_2_/5%CO_2_ and continuously perfused with oxygenated and preheated (33 ± 0.5 °C) artificial cerebrospinal fluid (aCSF) (in mM) [110 NaCl, 5 KCl, 2.5 CaCl_2_, 1.5 MgSO_4_, 1.24 KH_2_PO_4_, 10 D-glucose, 27.4 NaHCO_3_], perfused at 1.4 ml/min (electrophysiology); or ii) a recovery chamber with a modified aCSF medium, containing (in mM): 124 NaCl, 2.5 KCl, 2.5 CaCl_2_, 1.5 MgSO_4_, 1.25 NaH_2_PO_4_, 24 NaHCO_3_, 10 D-glucose, and saturated with 95%O_2_/5%CO_2_ for 1 h at 37 °C (biochemical assays).

### Electrophysiology

After 1.5 h incubation at 33 ± 0.5 °C in the recording chamber, a single glass pipette filled with 2 M NaCl was used to record field EPSPs (fEPSPs) elicited by stimulation of the Schaffer collateral pathway with twisted nichrome wires (single bare wire diameter, 50 µm) placed in CA1 stratum radiatum. Responses were recorded through a differential amplifier (DAM 50, World Precision Instruments, USA) with a 10 kHz high-pass and 0.1 Hz low-pass filter. Before each experiment, the input/output (I/O) relation was examined by varying the intensity of the stimulation. Long-term potentiation was induced using theta burst stimulation (10 bursts at 5 Hz, each burst consisting of 4 pulses at 100 Hz). Synaptic NMDA receptor-mediated responses were obtained using Mg^2+^-free aCSF containing 10 µM CNQX. Data were collected and digitized by Clampex; the slope of fEPSP was analyzed in most of the experiments, except for NMDA receptor-mediated responses in which the amplitude of fEPSP was analyzed. All data are expressed as means ± SEM, and statistical significance of differences between means was calculated with appropriate statistical tests as indicated in figure legends.

### Immunofluorescence of acute hippocampal slices

Hippocampal slices were collected 15, 40, or 60 min after TBS and fixed in 4% paraformaldehyde for 1 h and cryoprotected in 30% sucrose for 1 h at 4 °C, and sectioned on a freezing microtome at 20 μm. Sections were blocked in 0.1 M PBS containing 5% goat serum and 0.3% Triton X-100, and then incubated in primary antibody mixture including rabbit anti-SK2 (1:200) and mouse anti-PSD95 (1:200) and/or rat anti-Rab11 (1:200) in 0.1 M PBS containing 1% BSA and 0.3% Triton X-100 overnight at 4 °C. Sections were washed 3 times (10 min each) in PBS and incubated in appropriate Alexa Fluor–conjugated secondary antibodies for 2 h at room temperature. All images were taken in CA1 stratum radiatum between the stimulating and recording electrodes. All analysis and quantifications were performed as previously described^17^.

### P2/S2 fractionation, western blotting analysis, and immunoprecipitation

#### P2/S2 fractionation and Western blots

P2/S2 fractionation and Western blots were performed according to published protocols^17^. Briefly, hippocampus tissue or slices were homogenized in ice-cold HEPES-buffered sucrose solution (0.32 M sucrose, 4 mM HEPES, pH 7.4) with protease inhibitors. Homogenates were centrifuged at 900 g for 10 min to remove large debris (P1). The supernatant (S1) was then centrifuged at 11,000 g for 20 min to obtain crude membrane (P2) and cytosolic (S2) fractions. P2 pellets were sonicated in RIPA buffer (10 mM Tris, pH 8, 140 mM NaCl, 1 mM EDTA, 0.5 mM EGTA, 1% NP-40, 0.5% sodium deoxycholate, and 0.1% SDS). Protein concentrations were determined with a BCA protein assay kit (Pierce). The samples were separated by SDS-PAGE and transferred onto a PVDF membrane (Millipore). After blocking with 3% BSA for 1 h, membranes were incubated with specific antibodies overnight at 4 °C followed by incubation with secondary antibodies (IRDye secondary antibodies) for 2 h at room temperature. Antibody binding was detected with the Odyssey® family of imaging systems.

#### Immunoprecipitation

For immunoprecipitation, all procedures were carried out at 4 °C. COS-1 cells transfected with the indicated cDNAs and treated with either vehicle control or FSK/Ro (15-min incubation with 50 µM FSK plus 100 µM Ro 20–1724) were lysed with lysis buffer (Tris-HCl 25 mM pH 7.4, NaCl 150 mM, 1 mM EDTA, 1% NP-40, 5% glycerol and a protease inhibitor cocktail). After a brief centrifugation to remove insoluble material, the supernatant was precleared with an aliquot of agarose beads. For immunoprecipitation of Tac-SK2, extracts were incubated overnight with anti-Tac agarose beads, washed with lysis buffer, followed by elution of bound proteins by heating at 100 °C for 10 min in SDS-PAGE sample buffer. SDS-PAGE, Western transfer and immunoblotting were carried out as described above.

Immunoprecipitation of SK2 from hippocampal crude synaptosomal fractions was performed as previously described^17^, with minor modifications. Briefly, P2 lysates were incubated with anti-SK2 or anti-HA antibodies (negative control) coupled to Dynabeads protein G overnight at 4 °C, followed by four washes with wash buffer and elution in 2 X SDS sample buffer. Immunoprecipitated proteins were resolved by SDS-PAGE followed by Western blot analysis with specific antibodies against SK2, ubiquitin, and phosphoserine. At least three independent experiments were performed.

### Endocytosis assay of Tac-SK2 in COS-1 cells

Analysis of Tac-SK2 internalization was performed as previously reported^17,52^. In brief, **t**ransiently transfected COS-1 cells, plated on glass coverslips, were washed with ice-cold PBS, incubated with Tac7G7 antibodies for 1 h at 4 °C to label surface Tac, and transferred to the 37 °C incubator for 15 min to allow internalization. After fixation with 4% paraformaldehyde and 4% sucrose in PBS, cells were washed and incubated with Alexa 488-conjugated secondary antibodies to visualize the remaining surface Tac-SK2 proteins. Cells were then permeabilized with 0.25% Triton X-100 and internalized Tac-SK2 proteins were detected with an Alexa 594-conjugated secondary antibodies. For co-labeling with early endosome and late endosome/lysosome, cells were incubated with anti-EEA1 (1:500) and anti-LAMTOR4 (1:500) respectively overnight at 4 °C, and then incubated with Alexa Fluor 633–goat anti-rabbit IgG for 2 h at room temperature. After several washes, coverslips were mounted with Vectashield mounting media (Vector Laboratories, Burlingame, California) and analyzed with confocal microscopy.

### Images Analysis and Quantification

Cells were imaged using a Zeiss LSM 880 confocal laser-scanning microscope with a 60× objective. Images for all conditions in a particular experiment were obtained using identical acquisition parameters and analyzed using ImageJ software (NIH). All immunostaining studies were performed in 3–5 independent experiments. In all cases the experimenter was blind regarding the identity of the transfected constructs during acquisition and analysis. Quantification of endocytosis assay of Tac-SK2 was carried out as previously described^17^.

### Dynamics simulation of phosphorylated SK2

To gain insight on how phosphorylation affects the conformation of SK2, a homology model of SK2 C-terminal domain (residue ID 526 to 580) was developed using the *Molecular Operating Environment* (MOE)^53^. The model was used to prepare two MD simulation systems, SK2 C-term helix and a SK2 C-term helix with three phosphorylated serine residues (residue ID 568–570) using CHARMM-GUI^54,55^. CHARMM36 force field^56^ was used for all simulations. Each system was solvated into a rectangular water box consisted of CHARMM TIP3P water molecules^57^ and 150 mM KCl, with a box size of 136 × 136 × 136 Å^3^. All the MD simulations were performed using NAMD2.12^58^ under periodic boundary conditions at a constant temperature of 300 K and pressure of 1 atm (NPT ensemble)^59^. A smoothing function was applied to van der Waals forces between 10 and 12 Å. The solvated complexes were minimized and equilibrated using a stepwise procedure set up by CHARMM-GUI. Each system was run for 100 nanoseconds after equilibration steps.

### *In vitro* ubiquitination assay

GST-SK2 proteins containing the whole SK2 C-terminus and its S-D mutation were expressed in *E. coli* BL21 (DE3) and purified as previously described^17,36^. The E6AP (UBE3A) Ubiquitin Ligase Kit (Boston Biochem) containing E1 and E2 enzyme (UBE2D3), His_6_-UBE3A, ubiquitin, Mg^2+^-ATP, and reaction buffer was used. GST protein was used as a negative control. Three independent experiments were performed.

### Statistical analysis

Error bars indicate standard error of the mean. To compute p values, unpaired Student’s t test, and one- or two-way ANOVA with Tukey post-test were used, as indicated in figure legends.

## Supplementary information

Supplementary information.
