# Supplementary material for: PKA and Ube3a regulate SK2 channel trafficking to promote synaptic plasticity in hippocampus: Implications for Angelman Syndrome
Source: Sci Rep. 2020 Jun 17;10:9824. doi: 10.1038/s41598-020-66790-4 (PMC7299966; doi:10.1038/s41598-020-66790-4)
Supplement: Supplementary file 1 — Supplementary information. [file 41598_2020_66790_MOESM1_ESM.docx]

**Supplementary Information**

**PKA and Ube3a regulate SK2 channel trafficking to promote synaptic plasticity in hippocampus: Implications for Angelman Syndrome**

Jiandong Sun^1^, Yan Liu^2#^, Guoqi Zhu^2,4#^, Caleb Cato^1^, Xiaoning Hao^1^, Li Qian^3^, Weiju Lin^2^, Rachana Adhikari^1^, Yun Luo^3^, Michel Baudry^2^, Xiaoning Bi^1,*^

^1^ College of Osteopathic Medicine of the Pacific, ^2^ Graduate College of Biomedical Sciences, and ^3^ College of Pharmacy, Western University of Health Sciences, Pomona, CA 91766, USA, ^4^ Key Laboratory of Xin’an Medicine, Ministry of Education, Anhui University of Chinese Medicine, Hefei, 230038, China

^#^ Contributed equally to this work

Abbreviated title: **Phosphorylation facilitates SK2 channel ubiquitination**

***Corresponding author:** Dr. Xiaoning Bi

Dept. of Basic Medical Sciences, COMP

Western University of Health Sciences

701 E. Second Street

Pomona, CA 91766-1854

Tel: 909-469-5487

Fax: 909-469-5535

Email: [xbi@westernu.edu](mailto:xbi@westernu.edu)

**Full Original Blots**

Fig. 5c


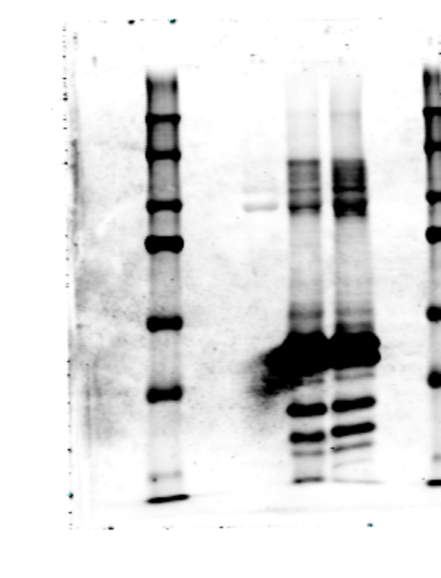

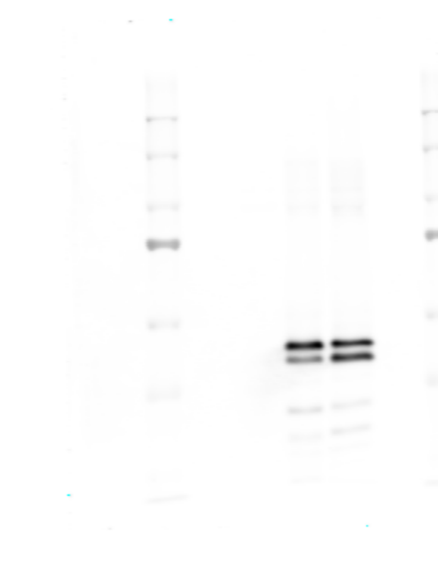


**50KD**

SK2

(original)

**50KD**

**250KD**

**150KD**

**100KD**

SK2

(enhanced exposure)

Fig. 5d


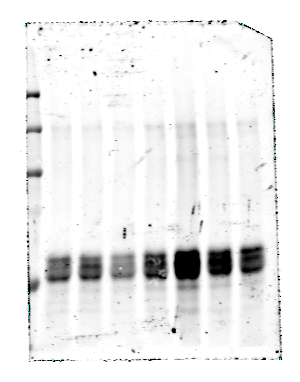

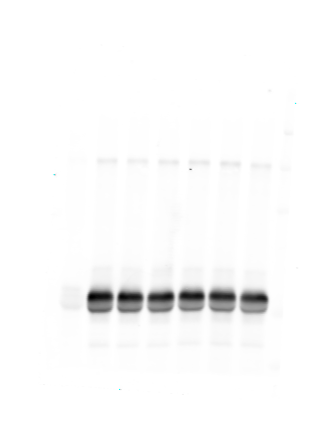


SK2

**50KD**

**250KD**

**150KD**

**100KD**

**75KD**

**50KD**

Ub

Fig. 6a


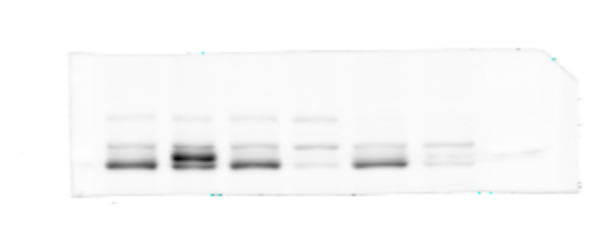

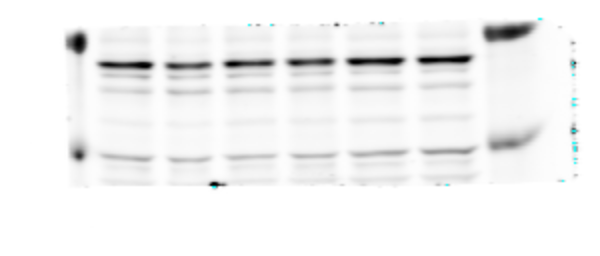

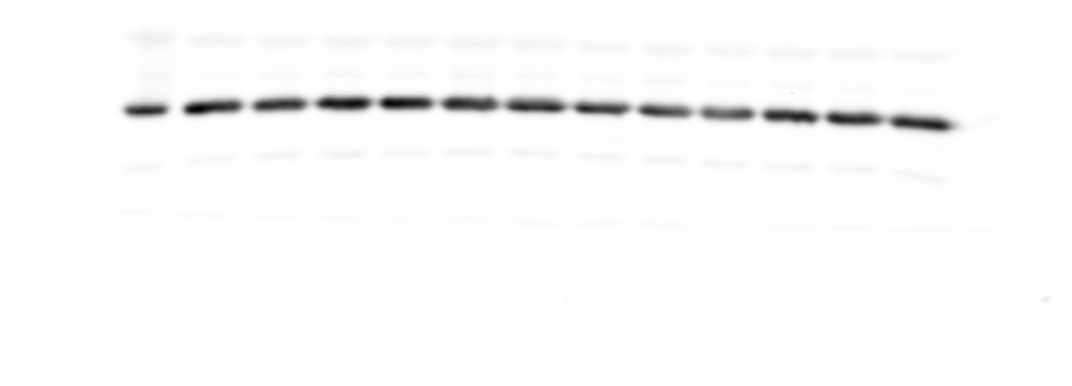


short

long

**50KD**

Ube3a

**100KD**

SK2

(alomone)

**75KD**

β-actin

**42KD**

Fig. 6c


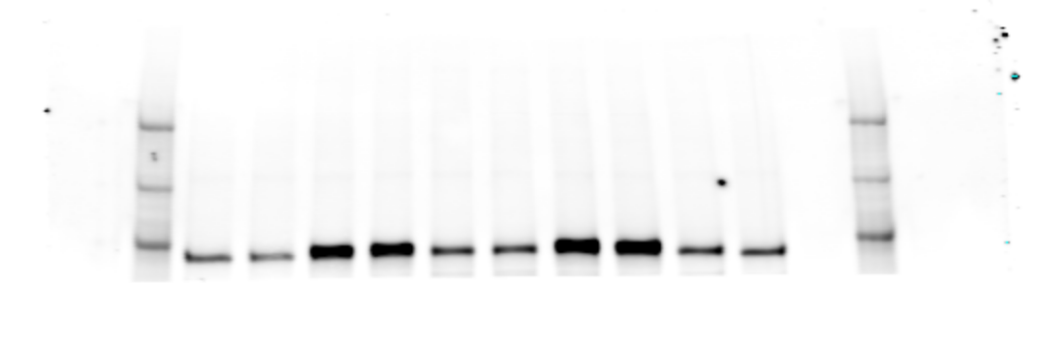

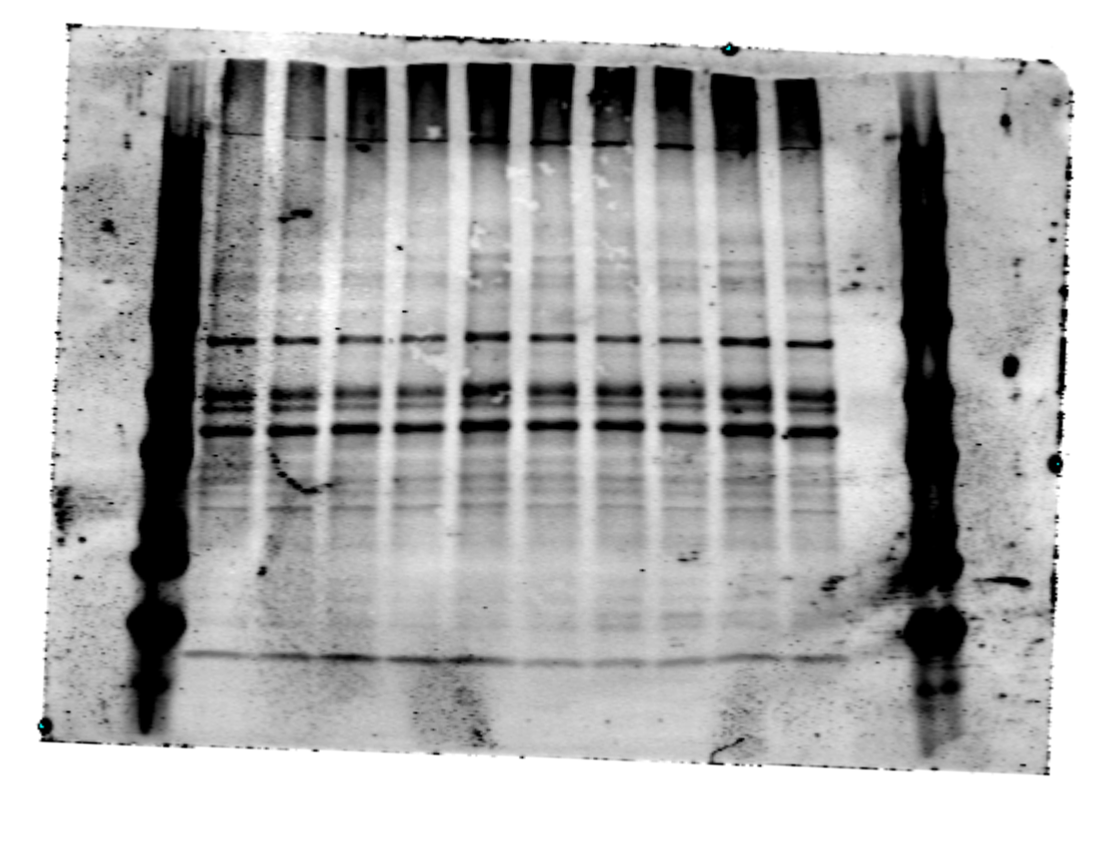


SK2

(alomone)

long

short

**75KD**

**50KD**

**100KD**

Ube3a


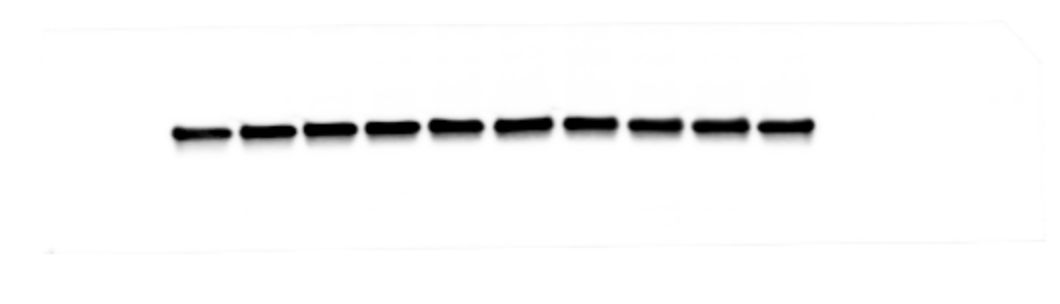


**42KD**

β-actin

Fig. 6e


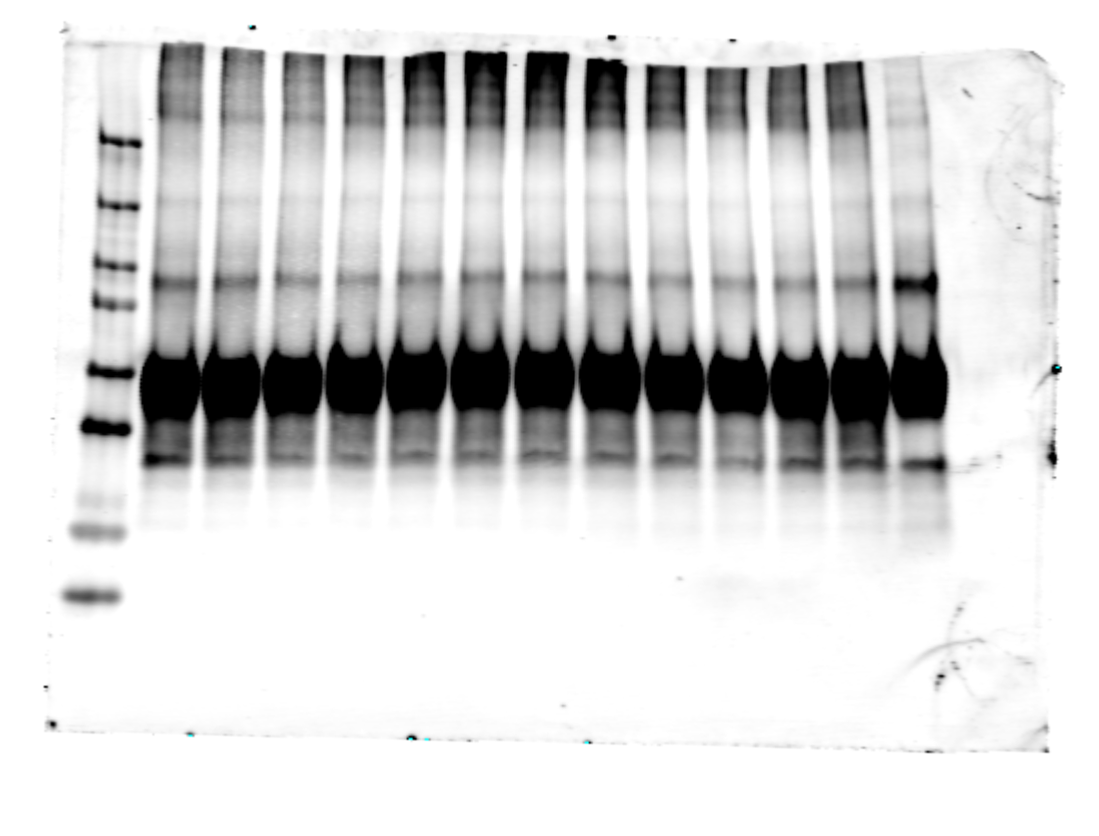

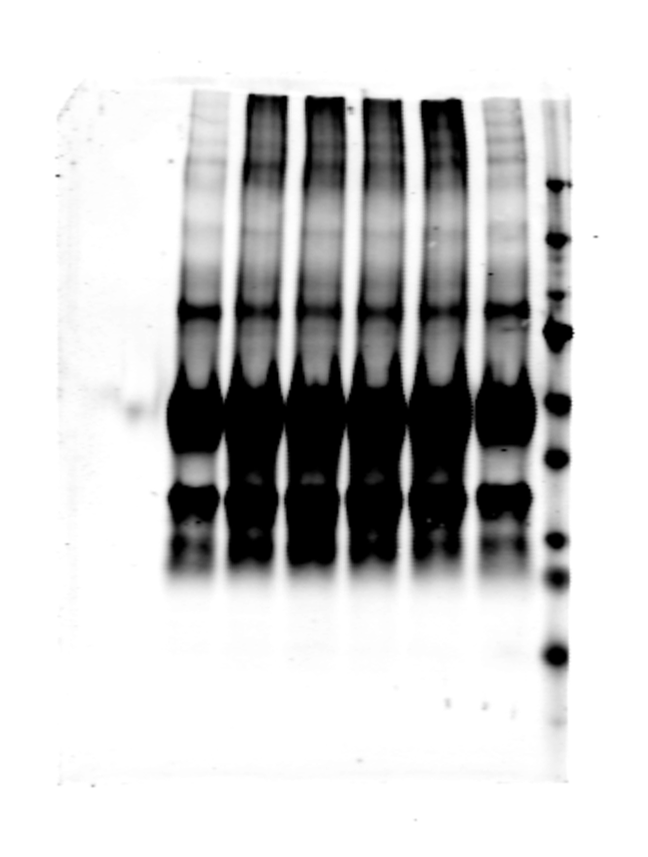

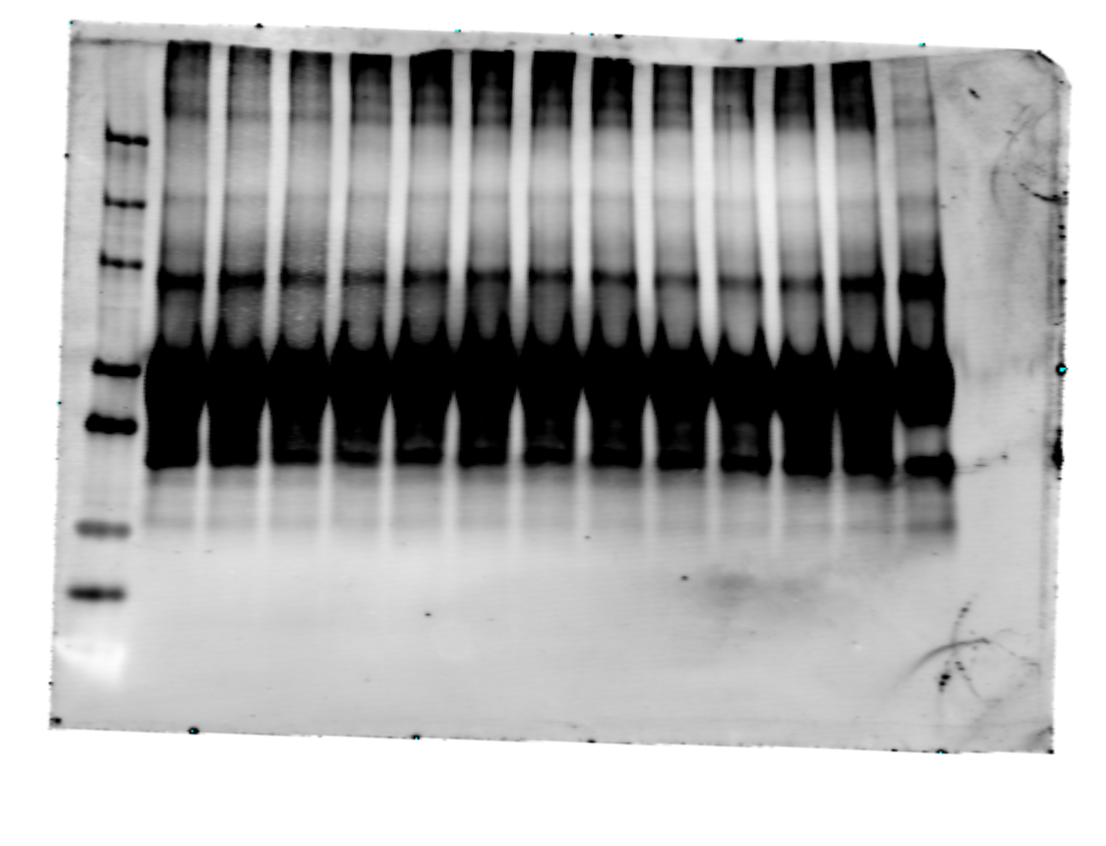


**75KD**

**100KD**

**150KD**

**250KD**

Ab heavy chain

Ub

phospho-serine

SK2

**Supplementary Figures**


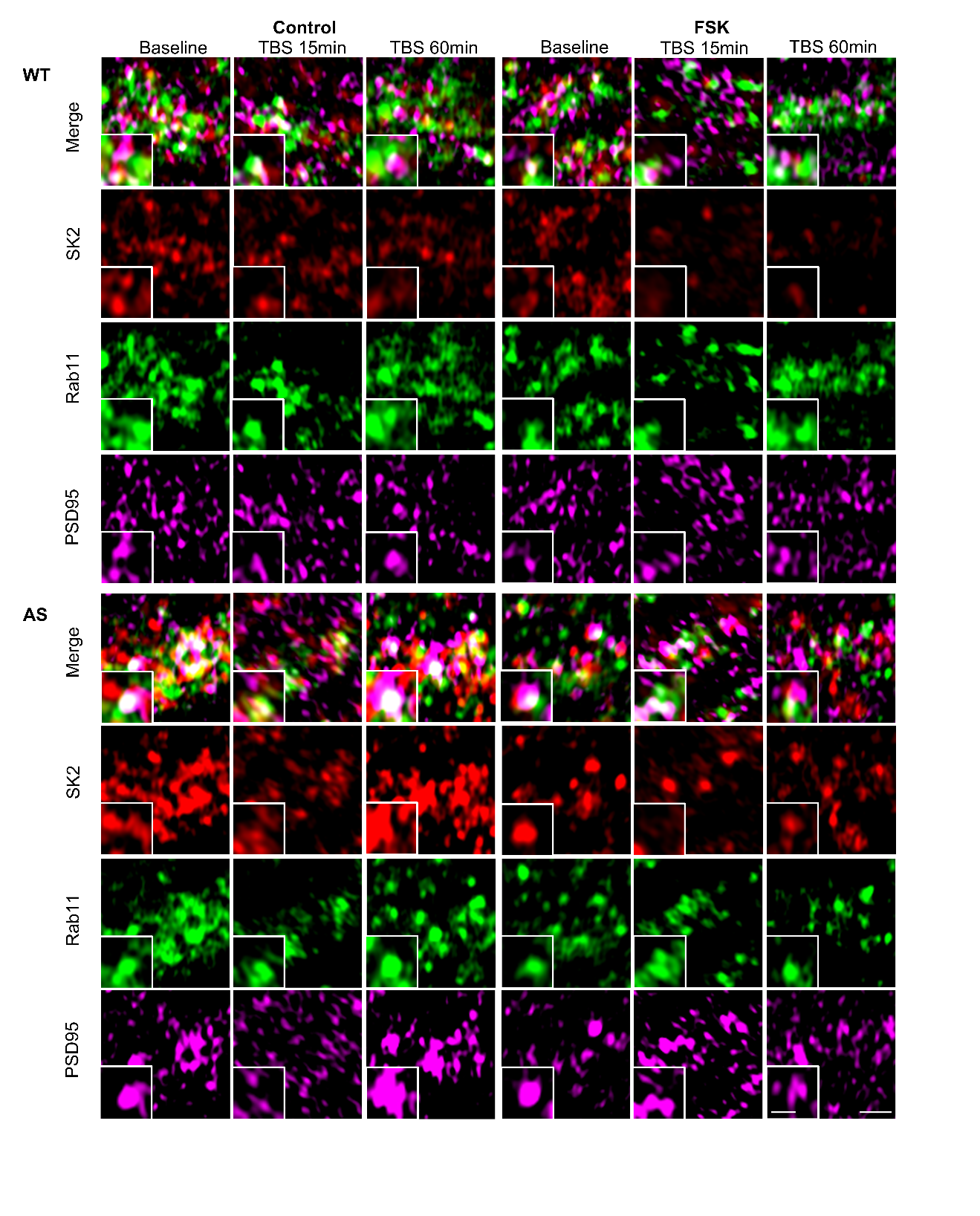


**Supplementary Fig. 1.** Representative images of SK2 (red), Rab11 (green), and PSD95 (magenta) co-immunostaining in hippocampal CA1 region of WT and AS mice at different time points after baseline recording (used as control) and TBS applications with or without FSK treatment. Scale bar, 1 µm, and 0.5 µm in insets. Related to Figure 4.


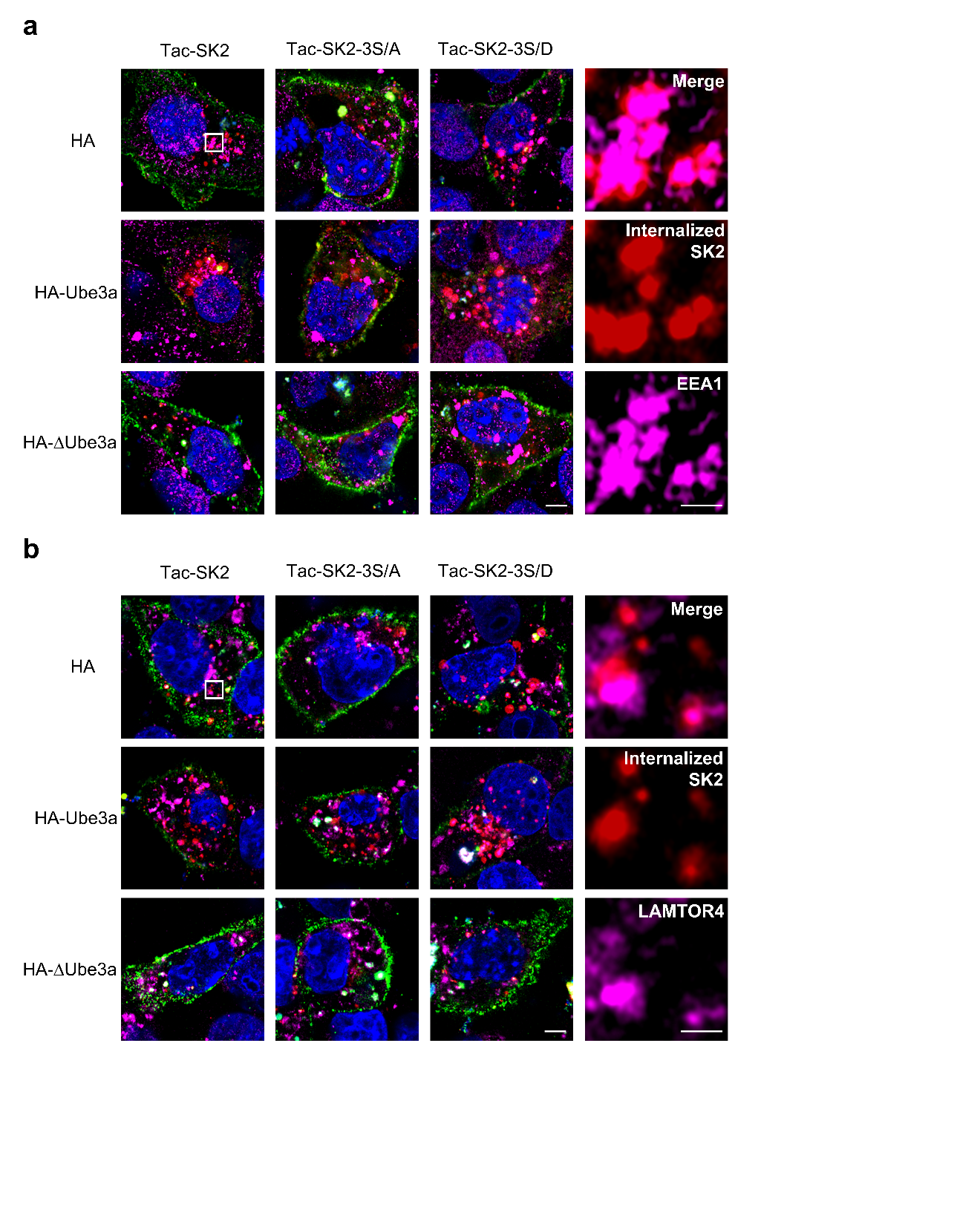


**Supplementary Fig. 2.** Effects of UBE3A overexpression and S-A or S-D mutations on SK2 surface expression and endocytosis, and co-localization of internalized SK2 with early endosome or late endosome/lysosome. Related to Figure 5.

**a.** Representative images of internalized (red) or surface-expressed (green) Tac-SK2, 3S/A, and 3S/D, and early endosome marker EEA1 (magenta) in COS-1 cells co-transfected with HA (top), HA-UBE3A (middle), or HA-∆UBE3A (bottom). Insets, co-localization of enlarged internalized SK2 and EEA1 puncta. Scale bar, 5 µm, and 1 µm in insets. **b.** Representative images of internalized (red) or surface-expressed (green) Tac-SK2, 3S/A, and 3S/D, and late endosome/lysosome marker LAMTOR4 (magenta) in COS-1 cells co-transfected with HA (top), HA-UBE3A (middle), or HA-∆UBE3A (bottom). Insets, co-localization of enlarged internalized SK2 and LAMTOR4 puncta. Scale bar, 5 µm, and 1 µm in insets.
